# Associated factors of secondary sex ratio of offspring in assisted reproductive technology: a cross-sectional study in Jilin Province, China

**DOI:** 10.1186/s12884-020-03373-1

**Published:** 2020-11-04

**Authors:** Mohan Wang, Xiangyin Liu, Han Zhang, Leilei Li, Ruizhi Liu, Hongguo Zhang, Yang Yu

**Affiliations:** Center of Reproductive Medicine, Center of Prenatal Diagnosis, the First Hospital, Jilin University, 71 Xinmin Street, Changchun, Jilin 130021 China

**Keywords:** Secondary sex ratio, Intracytoplasmic sperm injection, Assisted reproduction technology

## Abstract

**Background:**

The aim of this study was to determine the secondary sex ratio (SSR) of offspring in assisted reproduction technology (ART) in Jilin Province, China, and to analyse the influencing factors associated with SSR.

**Methods:**

A cross-sectional study of 3833 babies including singletons and twins born to 2990 couples treated by ART between May 2011 and December 2018 was performed.

**Results:**

The main outcomes of this study were that the SSR of ART babies in Jilin Province was 50.64% and the SSR was associated with fertilization methods *(p* < 0.05*)*. Comparing to in vitro fertilization (IVF), intracytoplasmic sperm injection (ICSI) (*OR = 0.808, 95%CI: 0.681–0.958*) decreased the percentage of male babies.

**Conclusions:**

This study suggests that the SSR of ART births in Jilin Province was lower than the normal level and ICSI had a significant effect on SSR. Though we need more samples to study in the future, we still need to think about the impact of ICSI on SSR in ART.

## Background

Human sex ratio is an important indicator of the gender balance of men and women. It is influenced by many factors. The ratio after conception is the primary sex ratio (PSR). And the ratio at birth is the secondary sex ratio (SSR). SSR is significantly lower than PSR due to abortion and many other factors. The normal value of SSR is approximately 100 females to 105 males [1]. The stability of SSR is of great significance for maintaining social stability and promoting economic development.

With the rapid development of assisted reproduction technology (ART ), more and more test-tube babies were born by ART [2]. It may have an effect on SSR compared to natural pregnancy. Gametes and embryos were exposed to external factors during ART, which might be different from the influence of internal factors on the SSR under natural pregnancy.Scholars began to study which factors in the process of ART will affect the SSR [3]. Most studies had demonstrated that the SSR in test-tube babies was influenced by the use of intracytoplasmic sperm injection (ICSI). Although this was not a definite conclusion, related studies showed a trend that ICSI would decreased the SSR comparing to in vitro fertilization (IVF) [4, 5]. Some researchers specifically used systematic review to clarify that blastocyst stage transplantation might increase the SSR when it was compared with cleavage stage transplantation [4–6]. Other aspects, single or multiple births might also have an influence on the SSR [7]. In addition to this, paternal age, maternal age, smoking, even stress were the influencing factors of SSR [8–10]. Environmentalists drew a conclusion that SSR was low in areas with high air pollution through research [10]. Thus it could be seen, the factors that could influence the SSR were complex.

There had been lots of studies showing the SSRs brought by ART and exploring the influencing factors of the SSR. Jilin Province as a large province in northern China, there were no research on this type had been reported. We used the data one of the large reproductive centre in the province to enumerate the SSR and try to analyse the influencing factors of it. This study will put forward referable suggestions for maintaining the gender balance in Jilin Province.

## Methods

### Study subjects

The data was collected from Centre for Reproductive Medicine, First Hospital of Jilin University, Changchun, China. The study subjects included babies born to 2990 couple patients who performed ART from May 2011 to December 2018. Oocyte donation cycles, pre-implantation genetic diagnosis cycles and pre-implantation genetic screening were not included. Our subjects included singletons and twins. One male baby and one female baby twins were excluded when we performed logistic regression analyses. And for multi-cycle couples, we only included the first delivery cycle. The study had been approved by the ethics committee of the First Hospital of Jilin University. And written informed consent was obtained from each participant.

### Definitions and measurements

SSR in this study was the ratio of male births to total births. The variables in the analyses included number of cycles, maternal age, paternal age, infertility duration, maternal BMI, infertility type, infertility factor, fertilization methods, embryo transfer morphology, embryo transfer stage and birth type. The number of cycles referred to the current cycle number of the patient’s delivery. Infertility duration referred to the number of years of infertility without contraception. Above two variables were all divided into groups by class interval. And the class interval of maternal age group and paternal age group were 10 years old. The maternal BMI was divided into three groups according to the World Health Organization criteria. And birth type included singletons and twins two groups.

### Data analysis

SPSS software ( Version 24.0, IBM SPSS, IBM Corp, Armonk, NY, USA ) was used to analyse the data. Univariate logistic regression analyses were performed to examine the correlations of various variables with SSR. Statistical significance was set to *p* < 0.10. The significant variables were put into multivariable logistic regression to estimate the influencing factors for SSR. *P* < 0.05 was considered to be significant.

## Results

A total of 3833 babies were included from 2990 mothers in this study. There were 1941 male babies and 1892 female babies. The total SSR of the sample was 50.64%. The SSR of singletons and twins were 50.12% and 51.30%, respectively. The SSRs of different infertility types, factors, methods and embryo transfer morphologies were different (see Table 1).

**Table 1:** SSRs of babies from different patients with ART and birth types

	Total births	Male babies	Female babies	SSR(%)
Infertility type				
Primary	2674	1361	1313	50.90
Secondary	1159	580	579	50.04
Infertility factor				
Male	1532	766	766	50.00
Female	1462	747	715	51.09
Both male and female	548	276	272	50.36
Unknown	291	152	139	52.23
Fertilization method				
IVF	2290	1177	1113	51.40
ICSI	1276	611	665	47.88
R-ICSI	267	153	114	57.30
Embryo transfer morphology				
Fresh	834	430	404	51.56
Thawed	2999	1511	1488	50.38
Birth type				
Singletons	2147	1076	1071	50.12
Twins	1686	865	821	51.30
Total	3833	1941	1892	50.64

Univariate logistic regression analyses showed that only paternal age and fertilization methods were associated with SSR (*p* < 0.10) in all variables .In terms of paternal age, the babies from highest paternal age group had a higher SSR than that from youngest age group (*p* < 0.10). And for fertilization methods, the SSR was significantly lower in ICSI group than IVF group (*p* < 0.05) ( Table 2).

**Table 2:** Univariate logistic regression analyses of different variables with SSR

Variables	Total births	Male babies	SSR(%)	OR	95%CI
Number of cycles					
1 ~ 3	2519	1272	50.50	1.000	
4 ~ 6	29	13	44.83	0.797	0.382–1.663
Maternal age group					
20 ~ 29	892	441	49.44	1.000	
30 ~ 39	1610	818	50.81	1.056	0.897–1.244
≥ 40	46	26	56.52	1.329	0.731–2.417
Paternal age group					
20 ~ 29	660	326	49.39	1.000	
30 ~ 39	1661	839	50.51	1.046	0.873–1.25
40 ~ 49	213	117	54.93	1.249	0.916–1.703
50 ~ 59	14	3	21.43	0.279	0.077–1.011
Infertility duration					
0 ~ 10	2433	1222	50.23	1.000	
11 ~ 20	115	63	54.78	1.201	0.825–1.748
Maternal BMI					
18.5 ~ 25	1723	876	50.58	1.000	
< 18.5	593	286	48.23	0.901	0.747–1.086
≥ 25	232	123	53.02	1.091	0.829–1.436
Infertility type					
Primary	1766	898	50.85	1.000	
Secondary	782	387	49.49	0.947	0.800-1.121
Infertility factor					
Male	987	483	48.94	1.000	
Female	989	510	51.57	1.111	0.931–1.325
Both male and female	368	183	49.73	1.032	0.812–1.311
Unknown	204	109	53.43	1.197	0.885–1.619
Fertilization method					
IVF	1544	800	51.81	1.000	
ICSI	822	380	46.23	0.800	0.675–0.947
R-ICSI	182	105	57.69	1.268	0.930–1.730
Embryo transfer morphology					
Fresh	656	341	51.98	1.000	
Thawed	1892	944	49.89	0.920	0.770–1.099
Number of embryos transferred					
1	214	117	54.67	1.000	
2	2288	1149	50.22	0.836	0.631–1.108
3	46	19	41.30	0.583	0.306–1.113
Embryo transfer stage					
Blastocyst	551	283	51.36	1.000	
Cleavage stage	1997	1002	50.18	0.954	0.790–1.152
Birth type					
Singletons	2119	1060	50.02	1.000	
Twins	429	225	52.45	1.102	0.895–1.356

Table 3 showed the results of multivariate logistic regression analysis. Fertilization method was the only significant factor influencing SSR (*p* < 0.05). ICSI was associated with SSR (OR = 0.808, 95%CI: 0.681–0.958) and decreased the SSR comparing to IVF.

**Table 3:** Multivariable logistic regression analysis of paternal age and fertilization methods with SSR

Variables	OR	95%CI	P
Paternal age group			
20 ~ 29	1.000		
30 ~ 39	1.020	0.850–1.223	0.833
40 ~ 49	1.220	0.894–1.665	0.209
50 ~ 59	0.298	0.082–1.079	0.065
Fertilization methods			
IVF	1.000		
ICSI	0.808	0.681–0.958	0.014
R-ICSI	1.261	0.924–1.721	0.143

## Discussion

The main findings of this study were as follows: the SSR of ART babies in Jilin Province was 50.64%; SSR was associated with fertilization methods. And comparing to IVF, ICSI might decrease the percentage of male babies.

SSR is often used as an important indicator of population health and fertility. As far we know, the normal value of SSR is 51.22% [1]. Different from those of large sample studies, an analysis from the UK showed that the SSR of babies born through ART was 50.98% [11]. It was lower than the normal value and approximated to ours. A study specifically on single embryo transfer revealed that the SSR of New Zealand and Australia were 51.3% and 51.5%, respectively [4]. They were all higher than the normal value. A Chinese large sample research [12] from 18 reproductive centers showed an SSR of 51.8%, it was beyond the normal level. From our data result and the national, we could preliminarily conclude that ART might influence the SSR in China. Due to some factors such as large sample size gaps, research time, geographical differences or social demography structure, our result was different from the above studies. Whether the relatively lower SSR of babies born through ART in Jilin Province will balance the total SSR of the province, including through natural pregnancy, remains to be studied.

Although our results only showed an association between fertilization methods and SSR, this result was consistent with the results of other similar studies [4, 5, 13, 14]. The development of ICSI was facilitated when researchers focused on male infertility by considering sperm factors at the beginning of treatment for infertility [15]. As second-generation IVF technology, ICSI has been widely used worldwide. But researchers have never stopped discussing the safety of ICSI [16]. In the China’s first large sample study [12] about SSR of ART babies, a preliminary analysis was conducted on the reasons why ICSI might reduce SSR. They thought that the patient’s spermatogenic function was impaired, normally functioning sperm with Y chromosome was also reduced. However, other studies had shown that patients with normal spermatogenesis had reduced SSR after ICSI [4]. We analyzed the possible reason was although the best indication for ICSI targeted male factors such as azoospermia, some patients had ICSI because of non-male factors [15]. For example, those patients who had previously experienced failed fertilization with IVF. Therefore, the above different research results might have different conclusions, depending on what the indications for ART were. In this study, male factors accounted for the largest proportion of infertility factors. The number of patients undergoing ART for both male and female factors also accounted for a significant proportion. And we separated patients who had ICSI from rescue-ICSI. Rescue-ICSI were all performed after unsuccessful IVF that were for non-male factors. Unlike with ICSI alone, no effect on SSR was shown when compared to IVF. Most infertile men in our centre were patients with azoospermia or chromosomal abnormalities. Therefore, we tentatively concluded that the proportion of male babies born to ART patients who were solely due to male factors was lower than that of other factors.

There are some limitations in our study. Firstly, our sample size is relatively small. As a newly developed technology in Jilin Province in recent years, the sample size was limited when the gender of the offspring was excluded as one male and one female. What’s more, the information we have collected is not comprehensive. We should collect information such as the test results of some reports from the parents, etc. This is the incomplete information caused by the incomplete electronic medical record system in the early stage. We think that in the future, with the gradual improvement of our system, we will analyse the impact on SSR from a more comprehensive perspective.

## Conclusions

In conclusion, we found that the SSR of assisted reproductive technology births in Jilin Province was lower than the normal level through this study. And ICSI had a significant effect on SSR. The use of ICSI decreased SSR comparing to IVF. Although we need more samples to study in the future, we still need to think about the impact of ICSI on SSR in the assisted reproduction technology.

## Data Availability

The data used in this study is not available for public view, but available from the corresponding author upon reasonable request.

## Abbreviations

SSR: Secondary sex ratio.
ART: Assisted reproduction technology.
IVF: In vitro fertilization.
ICSI: Intracytoplasmic sperm injection.
PSR: Primary sex ratio
